# Pharmacological Potential and Bioactive Components of Wild Anatolian Sage ( *Salvia aethiopis* L.)

**DOI:** 10.1002/fsn3.70118

**Published:** 2025-03-25

**Authors:** Emine Bilginoğlu, Hamit Emre Kızıl, Hatice Öğütcü, Güleray Ağar, Yavuz Bağcı

**Affiliations:** ^1^ Department of Field Corps, Faculty of Agriculture Kırsehir Ahi Evran University Kırsehir Türkiye; ^2^ Department of Medical Services and Techniques, Vocational School of Health Services Bayburt University Bayburt Türkiye; ^3^ Department of Biology, Polatlı Faculty of Science and Letter Ankara Hacı Bayram Veli University Ankara Türkiye; ^4^ Department of Biology, Faculty of Science Ataturk University Erzurum Türkiye; ^5^ Department of Pharmaceutical Botany, Faculty of Pharmacy Selcuk University Konya Türkiye

**Keywords:** antimicrobial activities, chemical characterization, cytotoxicity, *Salvia aethiopis* L.

## Abstract

*Salvia aethiopis* L., a member of the Lamiaceae family, has gained attention due to its bioactive compounds with potential therapeutic applications. This study investigated the chemical composition and biological activities of *S. aethiopis* ethanol extract collected from Karaman province, Türkiye. The phenolic profile of the plant was characterized using LC–MS/MS analysis, which revealed substantial amounts of rosmarinic acid, ferulic acid, caffeic acid, and p‐coumaric acid. The antimicrobial activity of the extracts was evaluated against various Gram‐positive and Gram‐negative bacteria, as well as fungal strains, using the agar well diffusion method. The results demonstrated potent antibacterial effects, particularly against *Staphylococcus aureus* , *Micrococcus luteus* , and *Klebsiella pneumoniae* . Furthermore, the cytotoxic potential of *S. aethiopis* was assessed on H‐460 non‐small cell lung cancer cells. The extract exhibited a dose‐dependent cytotoxic effect, with a significant reduction in cell viability after 24 and 48 h of treatment, yielding IC_50_ values of 80.08 and 56.19 μg/mL, respectively. These findings suggest that *S. aethiopis* possesses promising antimicrobial and anticancer properties, which could contribute to the development of novel therapeutic agents.

## Graphical abstract

The ethanol extract of *Salvia aethiopis* , collected from Karaman, was found to be rich in phenolic compounds through LC–MS/MS analysis, and it demonstrated potent antibacterial effects, particularly against *Staphylococcus aureus* , *Micrococcus luteus* , and *Klebsiella pneumoniae* , as well as a dose‐dependent cytotoxic effect on H‐460 lung cancer cells. These findings indicate that *S. aethiopis* has promising antimicrobial and anticancer properties that could contribute to the development of novel therapeutic agents.

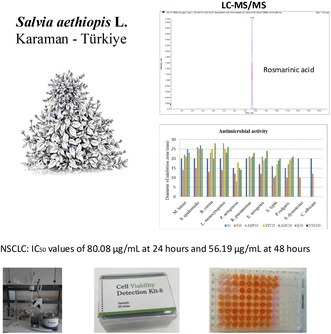

## 1 Introduction

Medicinal plant species have been used worldwide for seasoning and phytotherapeutic purposes, as food and beverages, and also in perfumes (Fan and Li 2023; Güven et al. 2023; Altay et al. 2022; Izol et al. 2021; Selek et al. 2018). This variety of uses is provided by the rich chemical components contained in medicinal plants. The Lamiaceae family is one of the important families of medicinal aromatic plants with functional properties (Moshari‐Nasirkandi et al. 2023). Many members of the Lamiaceae family are known for their secondary metabolites and bioactive compounds. Some species of this plant family, such as *Mentha spicata* , *Origanum onites* , *Hyssopus officinalis* , and *Salvia officinalis* , are used as spices, food additives, and flavorings in cuisine (Tahir et al. 2018). These plants are also traditionally used as antiseptics and in the treatment of asthma, fever, and cough, as well as for their carminative, tonic, and expectorant properties (Mahendran et al. 2021; Khazaie et al. 2008). Plants of the Lamiaceae family contain effective phytochemicals such as phenolic compounds, terpenoids, flavonoids, tannins, steroids, alkaloids, and saponins. These phytochemicals provide plants with antimicrobial, antioxidative, antiseptic, analgesic, and anti‐inflammatory properties (Kianmehr et al. 2024; Bozzini et al. 2023). Extracts obtained from different parts of plants through various methods provide valuable insights into their therapeutic potential. *Salvia*, an important genus belonging to the Lamiaceae family, comprises nearly 1000 different species (Uysal et al. 2023). *Salvia aethiopis* L., known as “woolly sage,” is native to Mediterranean countries such as Türkiye, Greece, Spain, Portugal, Italy, and Cyprus (Caylak 2024; Kellogg and Kubitzki 2015). *S. aethiopis* is a perennial plant with a height of 50–120 cm (Nurmahanova et al. 2023). Studies have been conducted on the morphological and anatomical characteristics of *S. aethiopis* , and the chemical compositions of its essential oil components in various ecosystems have been determined (Chalchat et al. 2001; Velickovic et al. 2003; Güllüce et al. 2006; Shahhoseini et al. 2024). Furthermore, based on the limited literature available on *S. aethiopis* , studies have investigated the isolation and purification of diterpenoids from its root extracts (Dekić et al. 2025). Medicinal and aromatic plants have been increasingly acknowledged as valuable sources of novel bioactive molecules with significant therapeutic potential (Ceylan et al. 2021). It is estimated that approximately 40% of all pharmaceuticals in use are derived, either directly or indirectly, from natural sources, with a substantial proportion originating from plant species (Malik and Upadhyay 2020; Boğa et al. 2016).

The aim of this study is to characterize the chemical components of *S. aethiopis* using the LC–MS/MS method and evaluate its biological activities. The antimicrobial effects of the *S. aethiopis* extract, with rosmarinic acid as its major component, were investigated in vitro against various bacterial strains. Additionally, the cytotoxic effects of different concentrations of the extract on human non‐small cell lung cancer (NSCLC) H‐460 cells and healthy lung fibroblast cells were examined over 24 and 48 h of incubation. In this context, the study aims to provide scientific data on the potential therapeutic effects of *S. aethiopis* , particularly its anticancer and antimicrobial properties.

## 2 Materials and Methods

### 2.1 Plant Material

*S. aethiopis* L. was collected during the flowering stage from Kazımkarabekir‐Karaman, Türkiye, at an altitude of 1010 m on July 6, 2022 (No. Bağcı 4224). The taxonomic identification of the plant material was conducted by plant taxonomist Yavuz Bağcı in the Department of Pharmacy, Selçuk University, Konya, Türkiye.

### 2.2 Preparation of the Extracts

The extraction procedure was adapted from Hernández‐Rangel et al. (2024) with minor modifications. Dried aerial parts of *S. aethiopis* were ground and extracted with 99% EtOH (Sigma, 1:8 w/v) using an orbital shaker at 110 rpm for 72 h. After filtration, the organic phases were concentrated under reduced pressure using a rotary evaporator (Scilogex, Rotavapor RE 100‐Pro, USA) at 40°C until dryness. The recovery of phenolic acids was achieved with a solvent of specific polarity. The obtained crude extracts were stored at −20°C for antimicrobial and cytotoxicity analyses.

### 2.3 LC–MS/MS Analysis

Secondary metabolite analysis of the EtOH extract from the aerial parts of *S. aethiopis* was performed using an Ultisil XB C18 column (2.1 × 50 mm, 5 μm) and AB Sciex 3200 QTRAP LC–MS/MS system. Molecules separated by LC–MS/MS according to their physicochemical properties were analyzed by mass detector. The instrument was operated in multiple reaction monitoring mode. The ionization source (ESI) and mass spectrometer parameters were optimized for highly accurate compound identification. The mobile phase consisted of water containing 0.2% formic acid and methanol containing 0.2% formic acid, with a flow rate of 0.3 mL/min. The analysis was conducted at 30°C (Yilmaz et al. 2022). The concentrations of analytes in the ethanol extract (mg analyte/g dry extract) are presented in Table 1.

**TABLE 1:** Phenolic compounds identified and quantified in the ethanol extract of *Salvia aethiopis* based on LC–MS/MS results (μg analyte/g dry extract).

	Analyte	RT	MI (m/z)	FI (m/z)	Ionization mode	μg
1	4 Hydroxybenzoic acid	7.16	136.8	134.0	Neg	ND
2	Caffeic acid	7.31	178.9	135.0	Neg	146
3	Rutin	7.32	358.8	161.0	Neg	ND
4	Hyperoside	7.39	462.9	271.0	Neg	ND
5	Ellagic acid	7.43	135.0	135.0	Neg	ND
6	Hesperidin	7.50	609.0	301.0	Neg	ND
7	p‐Coumaric acid	7.58	162.7	118.9	Neg	21
8	Rosmarinic acid	7.66	358.8	161.0	Neg	10,000
9	Syringic acid	7.89	196.8	123.0	Neg	230
10	3–4 Dimethoxycinnamic acid	7.9	209.0	191.1	Poz	ND
11	Luteolin	7.89	284.7	133.1	Neg	101
12	Kaemferol	7.90	286.9	153.2	Poz	ND
13	Quercetin	7.92	300.8	150.9	Neg	ND
14	Apigenin	8.06	271.0	153.1	Poz	37
15	Galangin	8.10	270.9	153.1	Poz	ND
16	Naringenin	8.15	270.7	119.0	Neg	ND
17	Chlorogenic acid	8.40	353.0	85.0	Neg	8
18	Ferulic acid	8.53	192.8	149.0	Neg	625
19	Genkwanin	8.61	282.9	268.0	Neg	135
20	Protocatechuic acid	9.20	152.8	108.8	Neg	10

Abbreviations: FI (m/z), fragment ions; MI (m/z), molecular ions of the standard analytes (m/z ratio); ND, not detected; RT, retention time.

### 2.4 Cell Viability Assay

The H‐460 (human NSCLC) cell line was obtained from the American Type Culture Collection (ATCC, USA). Cell proliferation was achieved in RPMI 1640 medium (Gibco, Thermo Fisher Scientific, USA) supplemented with 10% fetal bovine serum in 25 mL flasks. During cell passage, cells were washed with phosphate‐buffered saline (Merck KGaA, Darmstadt, Germany) and detached using trypsin–EDTA solution (Gibco). The flasks were maintained in an incubator at 37°C with 5% CO_2_ for 24–48 h. Cell viability was assessed before each passage using the trypan blue dye exclusion method (Merck KGaA). For cytotoxicity assessment, the CVDK‐8 kit (Ecotech Biotechnology, Erzurum, Türkiye) was used, which employs WST‐8, a tetrazolium salt that is reduced by cellular dehydrogenases to form an orange‐colored, culture medium‐soluble compound. This process relies on the principle that tetrazolium salts undergo reduction by accepting electrons from active mitochondria, resulting in formazan formation and a corresponding color change (Mosmann 1983). Since dead cells lack this ability and do not produce color changes (Riss and Moravec 2004), the amount of formazan dye produced directly correlates with the number of viable cells (Yildirim et al. 2022). Absorbance measurements were performed using a Multiskan GO Microplate Spectrophotometer (Thermo Fisher Scientific, USA).

### 2.5 Statistical Analysis

Data from three independent cytotoxicity experiments are presented as mean ± standard deviation. Statistical analysis was performed using GraphPad Prism software with Student's *t*‐test, and *p* < 0.05 was considered statistically significant.

### 2.6 Antimicrobial Activity

The antimicrobial activity was evaluated using the agar well diffusion method against various pathogenic microorganisms in triplicate. The test organisms included Gram‐positive bacteria ( *Micrococcus luteus* ATCC9341, *Staphylococcus epidermidis* ATCC12228, *Bacillus cereus* RSKK 863, *Listeria monocytogenes* ATCC19115), Gram‐negative bacteria ( *Salmonella typhi* H NCTC901.8394, *Proteus vulgaris* RSKK 96026, *Pseudomonas aeruginosa* ATCC27853, *Klebsiella pneumoniae* ATCC27853, *Enterobacter aerogenes* ATCC51342, *Shigella dysenteriae* NCTC2966), and yeast ( *Candida albicans* Y‐1200‐NIH). All strains were obtained from the Culture Collection of the Biology Department, Faculty of Science, Kırşehir Ahi Evran University (Nithya et al. 2011). Different concentrations of the *S. aethiopis* extract were prepared by dissolution in dimethyl sulfoxide (DMSO; Synth) followed by dilution to achieve a final DMSO content of 0.09% (v/v) and extract concentrations ranging from 4 to 0.007 mg/mL. DMSO solutions at the same concentrations served as negative controls. Positive controls included four antibiotics and one antifungal standard: ampicillin (AMP10, 10 μg), sulfamethoxazole (SXT25, 25 μg), kanamycin (K30, 30 μg), amoxicillin (AMC30, 30 μg), and nystatin (NYS, 100 μg) (Nartop et al. 2024).

## 3 Results and Discussion

### 3.1 Phytochemical Analysis by LC–MS/MS

*Salvia* (Lamiaceae) species are among the medicinal and aromatic plants that stand out for their rich composition, particularly in secondary metabolites. These plants are especially known for their high content of phenolic compounds, exhibiting antioxidant, antimicrobial, antiviral, antihemolytic, anti‐inflammatory, and cytotoxic activities (Kamatou et al. 2013; Lu and Foo 2002). Phenolic compounds play a significant role in plant defense mechanisms and have notable biopharmacological effects on human health (Dorman et al. 2003). Phenolic compounds represent a broad class of phytochemicals synthesized as secondary metabolites in plants, demonstrating properties such as antioxidant, antimicrobial, anti‐inflammatory, and neuroprotective effects (Cheynier 2012). These compounds are essential components of the plant defense system against abiotic and biotic stresses and are being extensively investigated for various biomedical and pharmaceutical applications (Dai and Mumper 2010). The classification of phenolic compounds primarily includes four main groups: phenolic acids, flavonoids, stilbenes, and lignans (Pandey and Rizvi 2009). These components exhibit potent antioxidant properties due to their ability to neutralize free radicals, playing a crucial role in preventing diseases associated with oxidative stress (Scalbert et al. 2005). Notably, phenolic acids (including rosmarinic acid, caffeic acid, and ferulic acid) and flavonoids (such as quercetin, luteolin, and apigenin) are biologically active compounds that enhance immune function, reduce inflammation, and provide protective effects against certain cancers (Haq et al. 2020; Manach et al. 2004). Regarding antimicrobial activity, phenolic compounds exert powerful effects on microorganisms by disrupting cell membranes, inhibiting enzymes, or affecting cellular DNA and protein structures (Cowan 1999). Specifically, *Salvia* and related Lamiaceae species demonstrate significant antibacterial and antifungal effects due to their high phenolic content, as supported by numerous studies (Dejene et al. 2024; Mourabiti et al. 2024; Bozin et al. 2007). Advanced analytical methods, particularly LC–MS/MS, play a crucial role in the qualitative and quantitative identification of phenolic compounds in *Salvia* species. This method provides detailed information about the plant's bioactive profile by detecting even trace amounts of components with high precision (Medina‐Holguín et al. 2008). The identification and quantification of phenolic compounds in *S. aethiopis* were performed using LC–MS/MS, providing a comprehensive profile of its bioactive constituents. As shown in Table 1, the analysis revealed the presence of several phenolic acids and flavonoids, with rosmarinic acid being the predominant compound (10,000 μg/g dry extract), followed by ferulic acid (625 μg/g), syringic acid (230 μg/g), and caffeic acid (146 μg/g). These compounds are well‐documented for their potent antioxidant, antimicrobial, and anti‐inflammatory activities, contributing to the pharmacological potential of *S. aethiopis* . The presence of luteolin, apigenin, and p‐coumaric acid, albeit in lower concentrations, further enhances the plant's bioactive profile. Notably, several phenolic compounds, including quercetin, rutin, and hesperidin, were not detected in the extract, possibly due to variations in extraction efficiency, plant chemotype, or environmental factors influencing secondary metabolite biosynthesis. The detailed LC–MS/MS profiling underscores the significance of *S. aethiopis* as a potential source of bioactive phenolics, warranting further investigation into its therapeutic applications.

In the ethanol extract of *S. aethiopis* , rosmarinic acid was found in the highest concentration, followed by ferulic acid, syringic acid, caffeic acid, genkwanin, luteolin, apigenin, p‐coumaric acid, and protocatechuic acid. The composition of secondary metabolites in medicinal plants varies according to geographical conditions and various environmental factors. Rosmarinic acid is a potent phenolic compound predominantly found in rosemary, sage, lemon balm, oregano, and basil. It has been isolated from 162 plant species across 26 different plant families (Trócsányi et al. 2020). Notably, the Lamiaceae family contains the highest number of rosmarinic acid‐rich plants, with 104 species. Within this family, the genus *Salvia* stands out as the most abundant in terms of rosmarinic acid content (Villegas et al. 2024). Rosmarinic acid exhibits various biological activities, including anti‐inflammatory (Luo et al. 2020), antiviral (Panchal et al. 2022), neuroprotective (Shang et al. 2017), antiangiogenic (Huang and Zheng 2006), antidiabetic (Ngo and Chua 2018), hepatoprotective (Elufioye and Habtemariam 2019), anticancer (Han et al. 2019), antifibrotic (Li et al. 2010), antioxidant (Muñoz‐Muñoz et al. 2013), and antidepressant (Kondo et al. 2015) effects. Ferulic acid content has been analyzed in four different *Salvia* species from various locations, with the following concentrations: *S. officinalis* (593.32 μg/g), *S. verbenaca* (9576.23 μg/g), *S. aegyptiaca* (519.01 μg/g), and *S. argentea* (4815.52 μg/g). Ferulic acid and its derivatives have been reported to provide numerous health benefits, including anticancer (Fahrioğlu et al. 2016), antimicrobial (Borges et al. 2013), anti‐inflammatory (Cunha 2010), and antidiabetic properties. Recent studies (Kalinowska et al. 2024) have demonstrated that p‐coumaric acid exhibits significant cytotoxic effects against various cancer cell lines. It showed particular efficacy in inhibiting HeLa cell growth with an IC_50_ value of 1 μg/mL and demonstrated significant cytotoxicity against HT‐29 cells with an IC_50_ value of 25 μg/mL. Furthermore, p‐coumaric acid showed antibacterial activity against *Staphylococcus aureus* , *Aspergillus niger*, *B. cereus* , *Escherichia coli* , and *Salmonella typhimurium* (Nasr et al. 2022; Bag and Chattopadhyay 2017). Both ferulic and p‐coumaric acids have garnered significant interest for applications in the food, health, cosmetic, and pharmaceutical industries due to their strong antioxidant activity. Additionally, p‐coumaric acid exhibits anti‐inflammatory (Pragasam et al. 2013), antioxidant (Roychoudhury et al. 2021), and antimicrobial (Boz 2015) properties. Onder et al. (2022) determined p‐coumaric acid concentrations through LC–MS/MS analysis in various *Salvia* species: *S. absconditiflora* (0.035 mg/g), *S. sclarea* (0.247 mg/g), and *S. palaestina* (0.069 mg/g).

### 3.2 Cell Viability

Non‐small cell lung cancer cells (H‐460) were treated with five different concentrations of *S. aethiopis* ethanol extract (200, 100, 50, 25, and 12.5 μg/mL) for 24 and 48 h. After 24 h of treatment, concentrations of 200, 100, and 50 μg/mL showed high cytotoxic activity, with a statistically significant increase in cell death (*p* < 0.05) and an IC_50_ value of 80.08 μg/mL. Although the same concentrations remained statistically effective after 48 h of treatment, cell death was observed to be substantially higher compared to the 24‐h treatment. The IC_50_ value obtained after 48 h of treatment was 56.19 μg/mL (*p* < 0.05) (Table 2).

**TABLE 2:** % Cell viability results of 24th and 48th hour treatment.

	24th hour	48th hour
	Mean ± SD	Mean ± SD
Control	100 ± 0.78	100 ± 0.79
200 μg/mL	6.408 ± 0.19*	8.528 ± 3.07**
100 μg/mL	70.095 ± 4.96*	14.186 ± 4.68**
50 μg/mL	75.978 ± 3.48*	72.652 ± 5.48**
25 μg/mL	110.310 ± 4.12	95.180 ± 2.60
12.5 μg/mL	121.050 ± 2.48	97.694 ± 3.50

*Note:* Mean: the average of the test results performed with three repetitions, % Cell viability: WST‐8 (CVDK‐8 kit) results. Data are presented as mean ± SD from three independent experiments. Statistical analysis was performed using Student's *t*‐test (GraphPad Prism), with *, ** means *p* < 0.05 considered significant.

Abbreviation: SD, standard deviation.

### 3.3 Antimicrobial Activity

The antimicrobial activity of *S. aethiopis* extract was examined against human pathogenic Gram‐positive and Gram‐negative bacteria and yeasts. Due to inappropriate antibiotic usage, numerous pathogens affecting human health have developed drug resistance, necessitating the discovery of novel compounds from natural sources, particularly plants. The extract demonstrated varying degrees of inhibitory effects (10–20 mm) against diverse pathogenic strains, showing moderate to high activities. The extract exhibited the highest activity against *S. epidermidis* , *M. luteus* , *B. cereus* , *L. monocytogenes* , *K. pneumoniae* , *S. dysenteriae* , and *C. albicans* . The antimicrobial activities of *S. aethiopis* extract concentrations were compared with four commercial antibiotics and one anticandidal agent (Table 3).

**TABLE 3:** Antimicrobial activities of different concentrations of *S. aethiopis* extract (diameter of inhibition zone [mm]).

Microorganisms	Concentrations										Positive controls				
	S1	S2	S3	S4	S5	S6	S7	S8	S9	S10	AMP10	SXT25	AMC30	K30	NYS100
*M. luteus*	20	20	20	20	16	15	15	15	15	14	22	21	25	23	N
*S. epidermidis*	20	20	15	15	15	15	15	15	15	15	26	25	27	25	N
*B. cereus*	20	20	18	18	17	16	15	13	13	13	23	25	20	28	N
*L. monocytogenes*	20	20	16	16	16	16	15	14	14	14	28	25	23	26	N
*P. aeruginosa*	15	15	15	15	14	14	14	14	12	12	8	18	15	14	N
*K. pneumoniae*	20	20	13	13	12	12	12	12	12	—	21	20	21	23	N
*E. aerogenes*	17	17	17	17	16	15	15	12	12	12	21	19	20	24	N
*S. typhi*	16	15	15	14	14	14	14	12	12	10	11	17	19	20	N
*P. vulgaris*	15	15	15	15	12	12	11	11	11	10	17	19	20	21	N
*S. dysenteriae*	20	20	15	13	13	13	12	12	11	10	—	—	—	—	N
*C. albicans*	20	20	17	17	15	15	15	15	12	12	N	N	N	N	20

*Note:* Concentrations of *S. aethiopis* extract: S1–S10; N: not tried; 1: ethanol; 2: DMSO. Standard reagents (diameter of zone inhibition [mm]): SXT25 (sulfamethoxazole 25 μg), AMP10 (ampicillin 10 μg), NYS 100 (nystatin 100 μg), K30 (kanamycin 30 μg), and AMC30 (amoxycillin 30 μg). Considered from three values.

**FIGURE 1:**
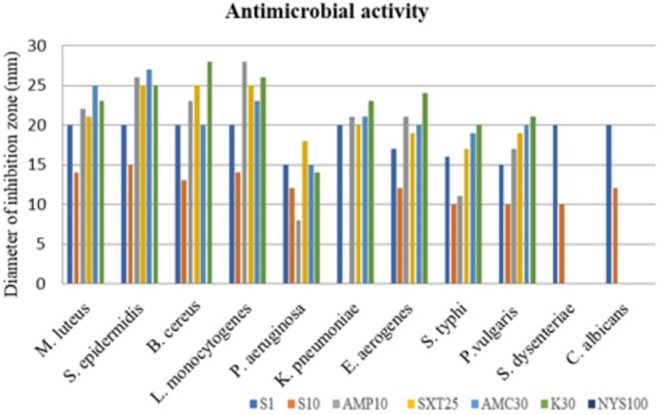
Antimicrobial activity of the highest (S1) and lowest (S10) concentrations of *S. aethiopis* extracts and standard reagents for Gram (+) and Gram (−) bacteria and yeast (standard reagents: AMP10, ampicillin [10 μg]; SXT25, sulfamethoxazole [25 μg]; AMC30, amoxicillin [30 μg]; K30, kanamycin [30 μg]; and NYS100, nystatin [100 μg]).

The extracts were initially dissolved in 5% DMSO at 800 μg/mL (highest concentration), followed by serial twofold dilutions ranging from 0.78 to 800 μg/mL (S1–S10). The antimicrobial activity data revealed that *M. luteus* showed highest inhibition zones with S1–S4 (20 mm), comparable to the commercial antibiotic SXT, while *B. cereus* demonstrated 20‐mm inhibition zones with S1 and S2, matching the commercial standard AMC (20 mm). All *S. aethiopis* extract concentrations (12–15 mm) showed superior inhibitory activity against *P. aeruginosa* compared to AMP (8 mm). S1–S4 (15 mm) exhibited higher antimicrobial activity than the K30 positive control, while S5–S8 (14 mm) showed equivalent activity. *K. pneumoniae* showed equivalent inhibition zones with S1 and S2 (20 mm) compared to SXT. While *K. pneumoniae* exists as normal flora in the mouth, skin, and intestines, it can cause severe pulmonary damage if aspirated (Podschun and Ullmann 1998). *E. aerogenes* showed highest inhibition with S1–S4 (17 mm), followed by S5 (16 mm) and S6–S7 (15 mm). For *S. typhi* , all extract concentrations except S10 (12–16 mm) demonstrated superior inhibition compared to AMP (11 mm). *Salmonella serovars* can cause various clinical manifestations, ranging from asymptomatic infection to severe typhoid‐like syndromes in infants or susceptible individuals. *S. dysenteriae* showed high inhibition with S1–S2 (20 mm), moderate inhibition with S3–S8 (15–12 mm), and low inhibition with S9–S10 (11–10 mm). *C. albicans* showed equivalent inhibition zones with S1–S2 (20 mm) compared to the anticandidal agent NYS, while other concentrations demonstrated moderate inhibition (17–12 mm). Overall, different concentrations of *S. aethiopis* extract demonstrated greater effectiveness against Gram‐negative bacteria compared to Gram‐positive bacteria (Table 3, Figure 1). This differential activity might be attributed to the presence of an outer impermeable membrane, thin peptidoglycan monolayer, periplasmic space, and distinct cell wall composition in Gram‐negative bacteria (Afzal et al. 2017; Ulular et al. 2024).

The Lamiaceae family has long been a focus of scientific interest due to its members' diverse biological activities, particularly their antioxidant, anticancer, and antimicrobial properties. *Salvia* species, an important genus within this family, have been extensively studied for their biological activities over many years. LC–MS/MS analysis of *S. aethiopis* revealed various phenolic compounds contributing to its biological activities. The study identified several key compounds, including rosmarinic acid, ferulic acid, caffeic acid, syringic acid, p‐coumaric acid, genkwanin, protocatechuic acid, apigenin, and chlorogenic acid, which are well‐documented flavonoids and phenolic acids with antioxidant, antimicrobial, and anticancer properties. *Salvia* species have garnered significant attention as potential cytotoxic agents due to their bioactive components. While previous studies on various *Salvia* species showed limited cytotoxicity against cancer cell lines (Luca et al. 2023), some notable exceptions exist. For instance, *S. officinalis* demonstrated promising anticancer effects in both animal models and various malignant cell lines, with sage tea consumption showing preventive effects against early‐stage colon carcinogenesis (Pedro et al. 2016). The plant's extracts exhibited proapoptotic and growth‐inhibitory effects against breast cancer, cervical adenocarcinoma, colorectal cancer, insulinoma, laryngeal carcinoma, lung carcinoma, melanoma, and oral cavity squamous cell carcinoma (Ghorbani and Esmaeilizadeh 2017). Regarding antimicrobial activity, our study demonstrated that *S. aethiopis* extract exhibited significant inhibitory effects against various pathogenic microorganisms. These findings align with previous research, though some variations exist in the literature. For instance, while Tohma et al. (2016) reported no antibacterial activity against certain strains, our study found notable inhibition zones comparable to standard antibiotics. These variations might be attributed to factors such as plant age, vegetation cycle stage, soil composition, and temperature, which can affect the quantity and quality of extracted compounds (Masotti et al. 2003; Angioni et al. 2006; Inci et al. 2023). The cytotoxicity results against the H‐460 lung cancer cell line are particularly noteworthy, as studies using *Salvia* species in lung cancer treatment are relatively scarce. While a previous study with *S. glutinosa* and *S. transsylvanica* against the A549 cell line showed modest effects (Mocan et al. 2020), our study demonstrated significant cytotoxicity with promising IC_50_ values after both 24‐ and 48‐h treatments.

## 4 Conclusion

This study provides valuable insights into *S. aethiopis* 's biological activities, demonstrating significant cytotoxic and antimicrobial properties supported by the presence of key bioactive phenolic compounds, particularly rosmarinic acid and ferulic acid. These findings highlight its potential as a source of novel therapeutic agents. However, further pharmacological testing and compound isolation studies are necessary to fully evaluate its clinical applicability in cancer and infectious disease treatments. The results not only contribute to our understanding of *Salvia* species' medicinal potential but also provide promising directions for future research and therapeutic development. The comprehensive analysis of both antimicrobial and cytotoxic activities, coupled with detailed phytochemical profiling, positions this study as a significant contribution to the field, while also highlighting the need for continued investigation into the mechanisms of action and potential therapeutic applications of *S. aethiopis* and related species.

## Author Contributions

**Emine Bilginoğlu:** conceptualization (equal), data curation (equal), formal analysis (equal), methodology (equal). **Hamit Emre Kızıl:** conceptualization (equal), data curation (equal), formal analysis (equal), investigation (equal), methodology (equal), writing – original draft (lead). **Hatice Öğütcü:** conceptualization (equal), data curation (equal), formal analysis (equal), investigation (equal), methodology (equal). **Güleray Ağar:** conceptualization (equal), project administration (equal). **Yavuz Bağcı:** conceptualization (equal), project administration (equal).

## Ethics Statement

All procedures performed in this study were in accordance with ethical standards. The study utilized the NCI‐H460 lung cancer cell line obtained from American Type Culture Collection (ATCC, USA) and did not involve direct human participants, human material, or human data collection.

## Consent

The authors have nothing to report.

## Conflicts of Interest

The authors declare no conflicts of interest.

## Supporting information


Appendix S1

## Data Availability

No datasets were generated or analyzed during the current study.
