# Correction to “Pharmacological Potential and Bioactive Components of Wild Anatolian Sage (*Salvia aethiopis* L.)”

**DOI:** 10.1002/fsn3.71201

**Published:** 2025-11-11

Kızıl, H. E., Bilginoğlu, E., Öğütcü, H., Ağar, G., & Bağcı, Y. (2025). Pharmacological Potential and Bioactive Components of Wild Anatolian Sage (Salvia aethiopis L.). Food Science & Nutrition, 13, e70118. 10.1002/fsn3.70118

In the originally published article, there was a typographical error in Table 2 (“% Cell viability results of 24th and 48th hour treatment”). In the row corresponding to the 24th‐hour treatment at the 100 μg/mL concentration, the value under the column “Mean ± SD” was incorrectly stated as “70.095 ± 4.96*”.

The correct value should have read: “40.095 ± 4.96*”.

This appears to be a simple keystroke error likely resulting from the close proximity of the “7” and “4” keys on the numeric keypad used during data entry.

This correction pertains only to this single numerical value within Table 2. Importantly, it does not change the reported statistical significance for this specific concentration.

Furthermore, this isolated correction does not alter the overall results, discussion, main findings, or conclusions presented in the article.

We apologize for this error.

