# Supplementary material for: Pharmacological Potential and Bioactive Components of Wild Anatolian Sage ( Salvia aethiopis L.)
Source: Food Sci Nutr. 2025 Mar 25;13(4):e70118. doi: 10.1002/fsn3.70118 (PMC11936837; doi:10.1002/fsn3.70118)
Supplement: Supplementary file 1 — Appendix S1 [file FSN3-13-e70118-s001.pdf]

SUPPLEMENTARY MATERIAL - LC-MS/MS Phenolic Compounds and Calibration Curves

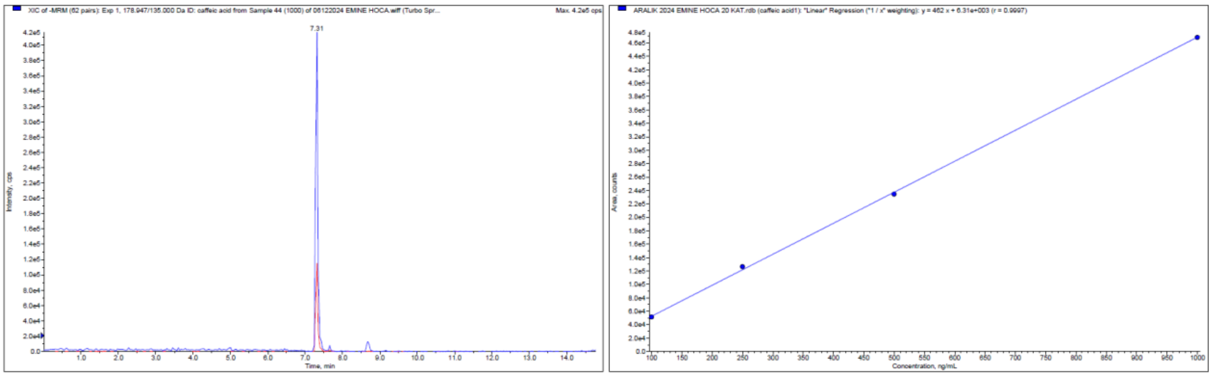

Figure 1. Caffeic acid

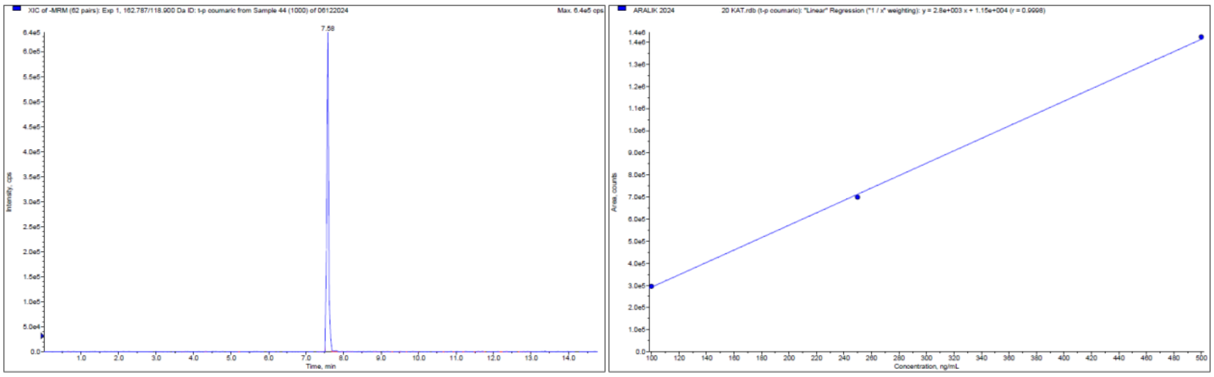

Figure 2. p-coumaric acid

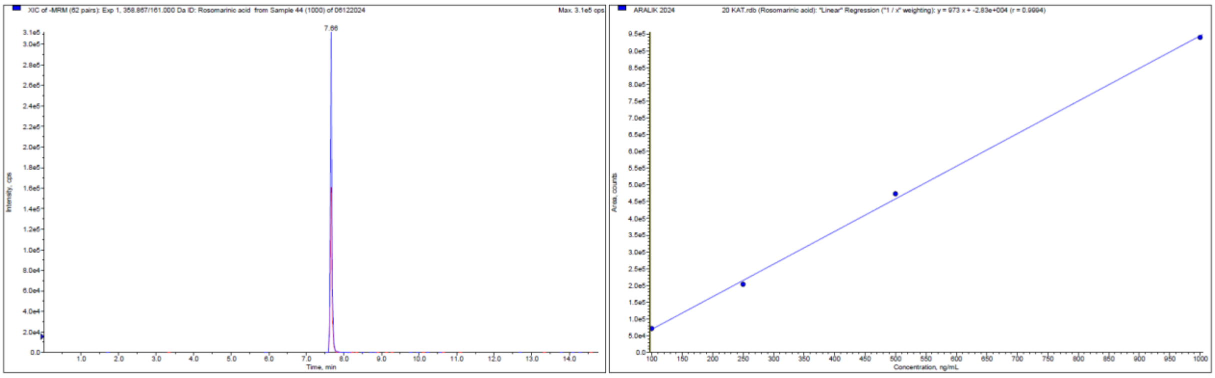

Figure 3. Rosmarinic acid

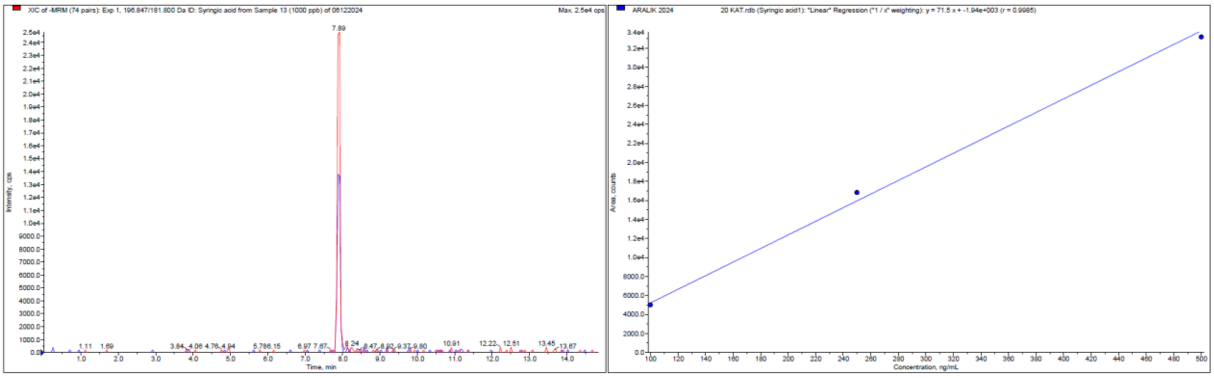

Figure 4. Syringic acid

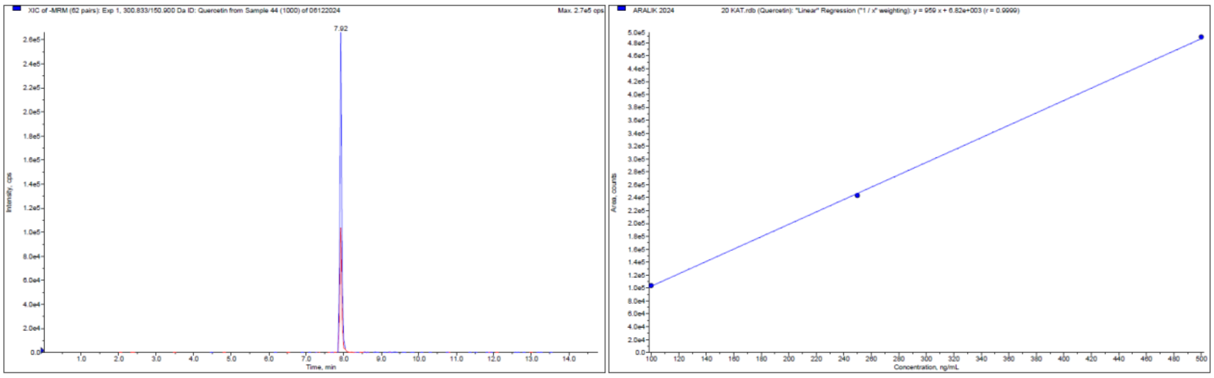

Figure 5. Quercetin

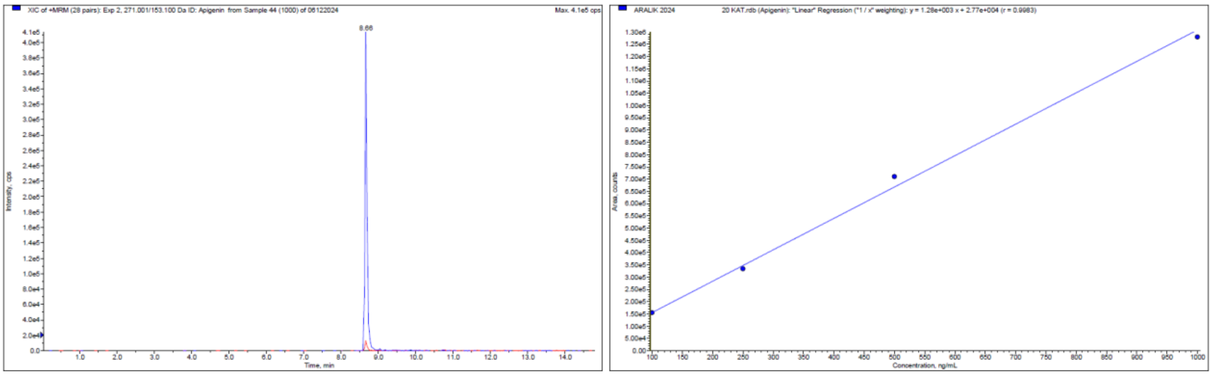

Figure 6. Apigenin

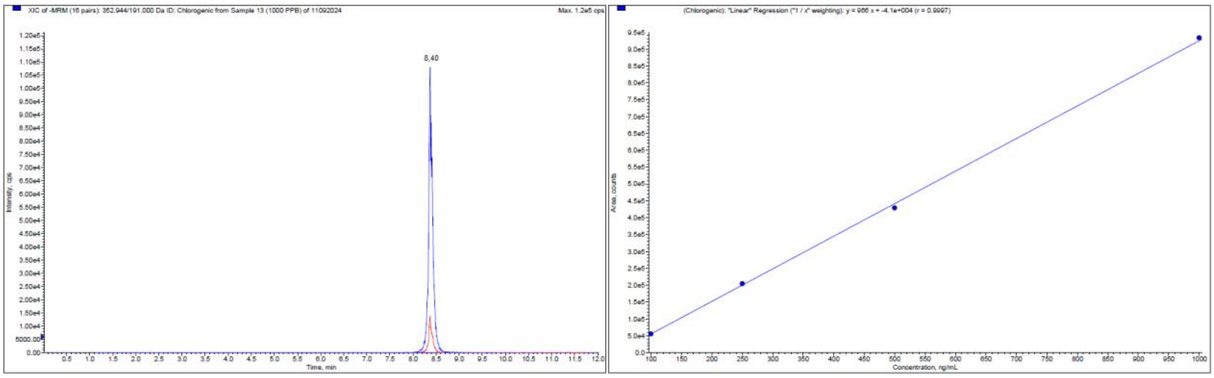

Figure 7. Chlorogenic acid

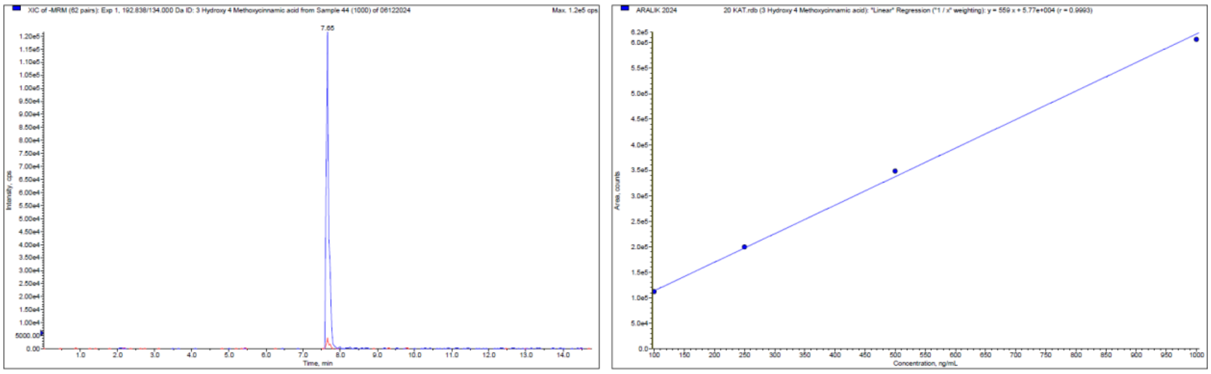

Figure 8. Ferulic acid

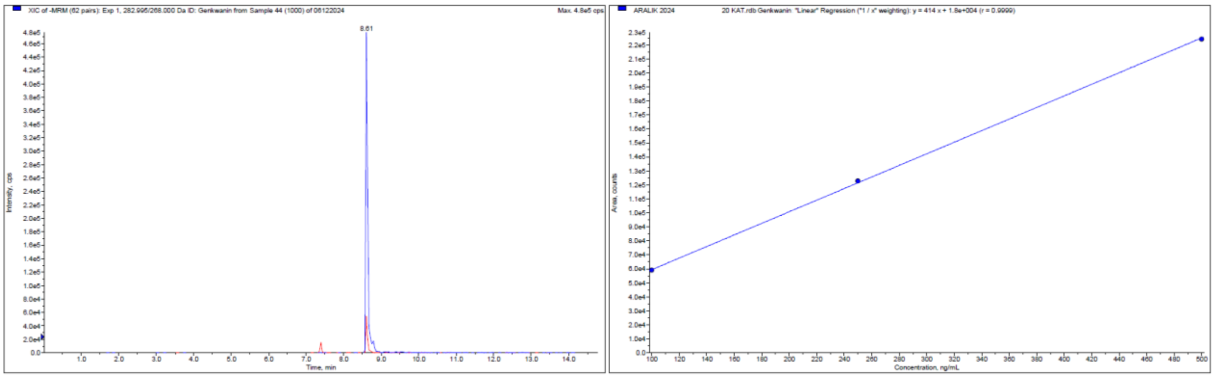

Figure 9. Genkwanin

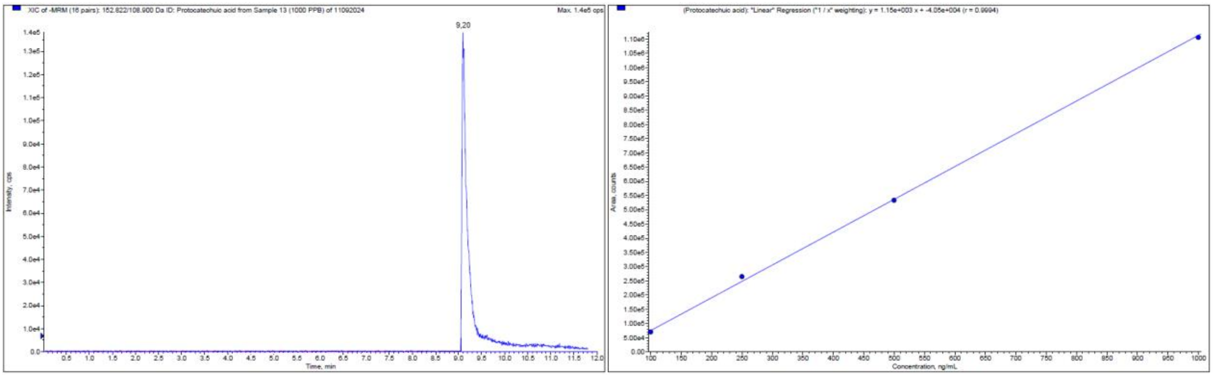

Figure 10. Protocatechuic acid
